# Liquid Biopsy Biomarkers in Urine: A Route towards Molecular Diagnosis and Personalized Medicine of Bladder Cancer

**DOI:** 10.3390/jpm11030237

**Published:** 2021-03-23

**Authors:** Matteo Ferro, Evelina La Civita, Antonietta Liotti, Michele Cennamo, Fabiana Tortora, Carlo Buonerba, Felice Crocetto, Giuseppe Lucarelli, Gian Maria Busetto, Francesco Del Giudice, Ottavio de Cobelli, Giuseppe Carrieri, Angelo Porreca, Amelia Cimmino, Daniela Terracciano

**Affiliations:** 1 Department of Urology of European Institute of Oncology (IEO), IRCCS, Via Ripamonti 435, 20141 Milan, Italy; matteo.ferro@ieo.it (M.F.); ottavio.decobelli@ieo.it (O.d.C.); 2 Department of Translational Medical Sciences, University of Naples “Federico II”, 80131 Naples, Italy; e.lacivita@studenti.unina.it (E.L.C.); antonietta.liotti@unina.it (A.L.); michele.cennamo2@unina.it (M.C.); 3 Institute of Protein Biochemistry, National Research Council, 80131 Naples, Italy; fabiana.tortora@ibbc.cnr.it; 4 CRTR Rare Tumors Reference Center, AOU Federico II, 80131 Naples, Italy; carbuone@hotmail.com; 5 Environment & Health Operational Unit, Zoo-Prophylactic Institute of Southern Italy, 80055 Portici, Italy; 6 Department of Neurosciences, Sciences of Reproduction and Odontostomatology, University of Naples Federico II, 80131 Naples, Italy; felice.crocetto@unina.it; 7 Department of Emergency and Organ Transplantation, Urology, Andrology and Kidney Transplantation Unit, University of Bari, 70124 Bari, Italy; giuseppe.lucarelli@inwind.it; 8 Department of Urology and Organ Transplantation, University of Foggia, 71122 Foggia, Italy; gianmaria.busetto@unifg.it; 9 Department of Urology, Sapienza University of Rome, 00185 Rome, Italy; francesco.delgiudice@uniroma1.it (F.D.G.); giuseppe.carrieri@unifg.it (G.C.); 10 Dipartimento di Oncologia ed Ematoncologia-DIPO-Università degli Studi di Milano, 20122 Milan, Italy; 11 Department of Urology, Veneto Institute of Oncology, 31033 Padua, Italy; angelo.porreca@gmail.com; 12 Institute of Genetics and Biophysics, National Research Council, 80131 Naples, Italy

**Keywords:** bladder cancer, liquid biopsy, free nucleic acids, urinary biomarkers

## Abstract

Bladder cancer (BC) is characterized by high incidence and recurrence rates together with genomic instability and elevated mutation degree. Currently, cystoscopy combined with cytology is routinely used for diagnosis, prognosis and disease surveillance. Such an approach is often associated with several side effects, discomfort for the patient and high economic burden. Thus, there is an essential demand of non-invasive, sensitive, fast and inexpensive biomarkers for clinical management of BC patients. In this context, liquid biopsy represents a very promising tool that has been widely investigated over the last decade. Liquid biopsy will likely be at the basis of patient selection for precision medicine, both in terms of treatment choice and real-time monitoring of therapeutic effects. Several different urinary biomarkers have been proposed for liquid biopsy in BC, including DNA methylation and mutations, protein-based assays, non-coding RNAs and mRNA signatures. In this review, we summarized the state of the art on different available tests concerning their potential clinical applications for BC detection, prognosis, surveillance and response to therapy.

## 1. Introduction

In the context of non-invasive methods in the diagnosis and follow-up of cancer, liquid biopsy is a valid alternative to tissue biopsy. Compared to the latter, in fact, liquid biopsy provides responses on tumor progression and therapeutic results in real time, as well as representing a non-invasive approach in disease diagnosis [1]. Liquid biopsy is also advantageous in terms of compatibility for real-time disease assessment [2]. For instance, radiological assessment approaches are unable to observe a low number of heterogeneous cancer cells. Therefore, a low sensitivity of evaluation is obtained, which results in a notable reduction in the chances of early diagnosis of the disease. Moreover, biopsy of tumor tissue, especially in the genitourinary (GU) system, can be inefficient and require invasive surgical options, especially for upper tract urothelial cancer. Liquid biopsy includes any non-tissue sample, especially body fluids, such as blood, feces, saliva, pleural fluid, peritoneal fluid, or brain spinal fluid, which may negate the need for costly, invasive, and sometimes painful tumor tissue biopsies for active surveillance of cancer. In particular, the discovery and characterization of some elements from urine, in recent years, has aroused considerable curiosity among scientific and clinical staff. Urine can turn out to be “liquid gold”, being the most abundant source of cancerous material [3]. Various methods are available for the isolation and characterization of these urinary components, which may involve genetic, and protein analyzes as well as microfluidic techniques. These are very specific and sensitive methods, advantageous from an operational point of view and in terms of processing speed and use of small sample volumes [4].

The three main used methods are ELISA to detect proteins, (rRT-PCR) for genetic material (DNA/RNA) and next-generation sequencing (NGS) which can assess the genomic sequence. Each of these diagnosis approaches has its own advantages and disadvantages. ELISA tests are sensitive and specific but can give false positive results. RT-PCR is a well-established methodology with high sensitivity, but it requires expensive consumables. NGS is highly repeatable and accurate, but it is expensive, and it needs high expertise and sophisticated laboratory equipment [5].

Scientific progress is undisputed in the use of urinary components as possible biomarkers for the diagnosis and surveillance of disease. These include exfoliated bladder cancer cells (EBCC), exosomes, and cell-free DNA (cfDNA).

Several types of cells are present in urine, namely epithelial cells, renal-derived cells, leukocytes, erythrocytes and urothelial cells, as well as genetic material, proteins, peptides and inorganic elements. Therefore, scientific and medical personnel prefer to use urinary biopsies for the potential diagnosis of diseases, such as colorectal cancer (CC) [4], bladder cancer [3], chronic renal disease [6], prostate cancer [7], cystic fibrosis [8] and chronic obstructive pulmonary disease [9]. Urine may be used in its entirety or divided into supernatant and pellet for biomarker analysis. The supernatant contains partially fragmented cell-free tumor nucleic acids and other cancer-derived materials, while the pellet mostly contains normal and exfoliated tumor cells, in addition to debris and possible bacteria. Recent studies have found that supernatant of urine is more useful than pellet for the analysis of genetic aberrations in patients with tumor of urothelial tract [10,11]. All biomarkers have their advantages and disadvantages, and their selection depends on the type of method available.

In the last few decades, cancer genetic profiles have been better characterized, providing potentially useful biomarkers for prognosis and prediction of drug response [12]. The use of urinary liquid biopsy in bladder cancer has gained growing attention [13]. In addition, molecular biology technologies have advanced to the point at which the application in clinical practice of technically demanding biomarkers will be soon even more possible. In this context, we provided an overview of the more recent studies regarding the emerging applications in clinical practice of the different biomarkers in urine-based liquid biopsy.

## 2. Methods

We performed an online search by PubMed/Medline using the terms “bladder cancer”, “non-muscle invasive bladder cancer” in combination with “urine biomarkers” and “liquid biopsy” limiting the search of articles published up to February 2021. This review included the original data of each clinical trial or observational study, which led to the development of urinary biomarkers. Two reviewers independently (M.F. and E.L.C.) selected articles, extracted data, removed duplicate publications. The inclusion criteria include: (1) urine-based biomarkers, (2) diagnostic biomarkers (3) biomarkers for the identification of disease recurrence. Only studies in English were included. The exclusion criteria include all conference abstracts, editorials, comments, letters to the editor and duplicates. The selection of literature is shown in Figure S1.

## 3. Biomarkers Used in Urinary Biopsy

**Figure 1:**
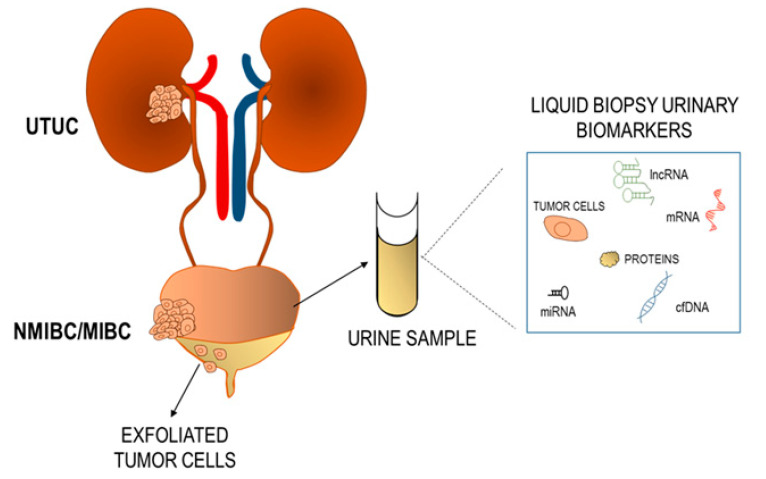
Overview of the liquid biopsy biomarkers in urine.

The analytes that can be used for tumor diagnosis in the liquid biopsy in urine setting include cell-free DNA (cfDNA), non-coding-RNA, exfoliated tumor cells, proteins (Figure 1).

Both healthy and diseased cells can release cfDNA during tumor destruction therapies or during apoptotic and necrotic processes. The cfDNA fragments, in most cases, are 100–200 base pairs in length. Because in physiological conditions the phagocytic cells englobe cellular debris and necrotic cells, we will have a very low cfDNA level in the disease absence [14]. On the other hand, in case of diseases, phagocytosis is not total, DNA digestion is minimal, and the DNA fragments have a random dimension that can even exceed 10,000 base pairs. As a result, the cfDNA level in the sick patient is elevated. Furthermore, cfDNA mutations give very important indications for the disease prognosis and diagnosis. CfDNA can be a useful biomarker derived from a urine biopsy. Indeed, scientists have discovered that cfDNA released by cells into the bloodstream can also be filtered at renal level and released in urine [15]. CfDNA can be analyzed in urine samples by ultracentrifugation or by molecular weight-based DNA separation techniques [16]. However, such a cfDNA extraction approach presents obstacles, as large fragments of cfDNA can escape capture, with consequent target DNA molecules loss or decrease. This can be overcome by processing a high number of samples. The extracted cfDNA can be amplified and analyzed using the various procedures that involve the polymerase chain reaction (PCR) and subsequent gene sequencing. Nevertheless, the frequency of observed mutations of cfDNA in urine often relies on the analysis technique and sample font. In fact, recent analysis found that the cfDNA level in urine is greater than that in the bloodstream [17]. Additionally, urine may be a circulating tumor cells (CTCs) source richer than blood, particularly in the case of renal, prostate, and upper and lower urothelial tract carcinoma, because urine wets these genitourinary organs. Further advantages of urinary biopsy concern the ease of acquisition, in fact there is no need for qualified medical personnel, less inconvenience for the patient, the use of a very small sample volume and mostly the urine contains fewer contaminating proteins than blood. The proteins biodistribution in the human body is based on the protein’s hydrodynamic diameters. In case of renal filtration, proteins with a diameter of less than 5 nm will be excreted rapidly in the urine, while proteins with a diameter greater than 15 nm will be retained by kidney filtration [11]. Glomerular filtration rate was assessed by endogenous small molecule such as urea and creatinine or smaller (less than 66 kDa) as cystatin C and β2-microglobulin [18].

An abnormal increase in specific proteins in the patient’s urine can be a marker of disease and has been proposed as biomarker for molecular detection, surveillance and prediction of targeted therapy efficacy [19].

Recently, preliminary studies on urinary peptide profiles as part of prognostic model to assess the risk of disease recurrence were available [20,21]. However, the potential clinical impact of these models needs to be further assessed in clinical trials on larger population.

In addition, it has been currently characterized the association of urinary microbiome and bladder cancer as well as the different urinary microbial profiles between non-muscle invasive bladder cancer (NMIBC) and muscle invasive bladder cancer (MIBC) [22,23]. These findings will probably pave the way to the identification of new biomarkers.

## 4. Molecular Detection and Surveillance

Urine can be collected at the time of the onset of symptoms related to urothelial carcinoma such as micro- and macro-hematuria or low-urinary tract manifestations.

European Association of Urology (EAU) guidelines recommend execute cystoscopy in patients presenting with hematuria [24], however, bladder cancer (BC) was found in a percentage of cases lower than 30% [25]. Therefore, a lot of unnecessary cystoscopies were performed with an increase of the cost of the whole diagnostic-therapeutic pathway and a relevant impact for the patient undergone to invasive procedure [26]. Urinary biopsies allow to avoid invasive cystoscopy or defer it. Several different new tests have been developed with a clear tendency towards the use of combination of biomarkers (Table 1).

## 5. DNA Methylation

### 5.1. Bladder EpiCheck

DNA methylation pattern changed during progression from NMIBC to MIBC [27]. Changes in DNA methylation pattern of a panel of 15 genomic biomarkers detected by RT-PCR was used in the Bladder EpiCheck (Nucleix, Rehovot, Israel) [28]. The test provides a score, which is considered positive for values higher than 60 [29]. Several reports were available on the diagnostic performance of Bladder Epicheck. Collectively, these studies indicated that Bladder Epicheck showed higher sensitivity particularly for low-grade cancer compared to cytology, whereas specificity was higher for cytology [29,30,31,32].

### 5.2. Urodiag

Since it has been demonstrated that high-risk tumors in general have high amount of hypermethylated genes than low-risk tumors [33], Roperch and Hennion [34] proposed a model combining a panel of FGFR3 mutations and a set of epigenetic markers (hypermethylation of the *HS3ST2*, *SEPTIN9* and *SLIT2*). Such a model in a study population including 263 subjects showed a very high diagnostic accuracy for the monitoring of BC patients with sensitivity/specificity/Negative predictive value (NPV)higher than 95%/76%/99%, respectively [35]. On this basis, the authors designed the Urodiag^®^ PCR Kit that includes an easy-to perform, fast and low-cost (~$100 per sample) tool for molecular detection and personalized surveillance of NMIBC.

### 5.3. AssureMDX

AssureMDX is a urinary combining DNA methylation and mutations. The test is based on the study of methylation of OTX1, ONECUT2 and TWIST1 genes and mutations in FGFR3, TERT, and HRAS genes [36]. Two different multicenter studies (including 154 and 977 patients respectively) showed a potential diagnostic utility of the test with a pooled sensitivity of 0.95 [95% CI 0.87–0.98] and specificity of 0.85 [95% CI 0.79–0.89] [26,37].

## 6. DNA Mutations

### 6.1. UroMuTERT

Telomerase reverse transcriptase (TERT) promoter mutations (C228T and C250T) occur in about 60–90% BC [38,39]. It is possible to detect these mutations in the urine of BC patients, providing a great opportunity for an easy diagnostic test for detection and surveillance of BC patients.

Avogbe et al. developed a test based on Next-Generation Sequencing called UroMuTERT for the comprehensive analysis of urine cell pellet and cell-free DNA in BC patients [40]. The authors demonstrated that the detection of TERT promoter mutations in urine have high diagnostic accuracy and excellent diagnostic specificity for BC [40,41]. More recently, Hosen et al. reported the development of test based on droplet digital PCR able to detect TERT promoter mutations in urine with an easy, inexpensive, non-invasive procedure [42]. Such an assay showed comparable performance with previously developed NGS-based test [40] but has the potential to be implemented in large-scale clinical application. Furthermore, TERT promoter mutations were revealed in urine 10 years before BC diagnosis and were not detected in healthy subjects in a recently published prospective study [41]. These findings suggested that urinary TERT promoter mutations could be a promising non-invasive biomarker for BC detection and surveillance.

### 6.2. Uromonitor

Mutations in fibroblast growth factor receptor 3 (FGFR3) are reported in about 35% BC [88]. The mutations G372C, R248C, S249C, and Y375C were analyzed in the urine and then evaluated as a tool for the diagnosis and monitoring of BC patients [89,90]. Unfortunately, two clinical studies including 772 and 97 patients respectively showed low sensitivity on the detection of high-grade tumors [43,91].

Recently, a new urine test called Uromonitor-V2^®^ (U-monitor, Porto, Portugal) was developed [44]. This test combines a group of hotspot mutations in three genes (TERT, FGFR3, and KRAS), among the most common genetic alterations in BC by RT qPCR.

Uromonitor-V2^®^ is based on a widely used technology and equipment and represent a low-cost and short time response test. However, this test was evaluated in a small number of patients with histologically confirmed recurrence and needs further studies on larger population of high-grade NMIBC patients.

### 6.3. Urovysion

Several authors showed that a test based on the fluorescence in situ hybridizations called UroVysion^®^ can help to detect urothelial carcinoma of the upper urinary tract better than urine cytology [45,46,47,48]. This test relies upon the analysis of chromosome aneuploidy on exfoliated urothelial cells collected from urine and allows to detect disease progression in Bacillus Calmette-Guérin (BCG)-treated patients [49]. However, genetic alterations can be detected in urinary cfDNA by droplet digital PCR and NGS methods with a significantly higher sensitivity [92].

### 6.4. Uroseek

Uroseek^®^ is an assay designed to reveal common alterations in BC of the following 11 genes: CDKN2A, HRAS, ERBB2, MET, FGFR3, PI3K, TP53, TERT, KRAS, MLL and VHL [50]. Eich et al. analyzed 527 cases and reported that the majority of studied cancers have at least one of the mutations of the panel, suggesting the strengthen and potential clinical benefits of the test [51].

### 6.5. uCAPP-Seq

Recently, a study from Stanford University including 118 BC patients and 67 healthy subjects demonstrated that a sequencing-based method for urinary tumor DNA detection, uCAPP-Seq, had a sensitivity of 84% and a specificity of 96–100% in BC detection [52].

## 7. Protein-Based Assays

### NMP22 and Basement Membrane-Derived Antigen (BTA)

The only two protein-based urinary assays FDA-approved and CE-marked are NMP22^®^ and BTA^®^, recommended in combination with cystoscopy for surveillance, but not for initial diagnosis because of false positive results due to hematuria, stones, and infectious disease and endoscopic procedures [93].

NMP22 is a nuclear matrix protein, abundantly expressed in malignant urothelial cells and released in urine by dead cells [53]. Both a quantitative ELISA assay and a qualitative test were available targeting NMP22. In a systematic review of the clinical benefit of urinary biomarkers, NMP22 assays showed a pooled sensitivity of 69% (CI 50–85%) and pooled specificity of 81% (46–93%) [54]. More recently, it has been highlighted a significant variation among studies and in some cases very low sensitivity values [55]. At present, NMP22 test is not widespread adopted in clinical practice even if still recommended in EAU guidelines in case of negative cystoscopy for patients at high-risk of recurrence [94].

BTA is a basement membrane-derived antigen released by cancer cells [56]. Two BTA assays are available: the ELISA BTA-TRAK-assay (Bard Diagnostics, Redmont, USA) and the qualitative test BTA-STAT (Bard Diagnostics, Redmont, USA) [57,58].

As NMP22, BTA assays are approved for surveillance in addition to cystoscopy, but not for diagnostic scope. Unfortunately, despite sensitivity values higher than cytology [55], BTA tests are not routinely used in clinical practice because of the high rate of false positive results [59].

## 8. Adxbladder

ADXBLADDER is an ELISA test measuring the urinary level of MCM5 (minichromosome maintenance protein 5). MCM5 belongs to a family of proteins playing a prominent role in DNA replication [60]. These proteins are overexpressed in highly proliferating cancer cells compared to healthy urothelial cells [61]. There is evidence that MCM5 amount was significantly associated with bladder tumor aggressiveness [62]. Several authors evaluated the potential clinical benefit of MCM5 as urinary biomarker [63,64,65,66,67]. ADXBLADDER is an easy to perform and cheap ELISA test, but still few data were available on the clinical benefit of its use in clinical practice. Dudderidge e al [65] in a prospective study including 856 subjects with hematuria showed a sensitivity of 73% (CI 61–83%) and a NPV of 96% (95–98%) for all cases, 86% and 99% for high grade cancers, 55% and 98% for low grade cancers, respectively. The findings suggested that ADXBLADDDER could be more sensitive than cytology at initial diagnosis of BC in patients with hematuria. In a smaller set of patients (n = 91, with 40 confirmed cancer cases) lower values of sensitivity (60%) and NPV (74%) was reported [66]. In a series of 1431 patients with NMIBC undergoing cystoscopic surveillance, Roupret et al. demonstrated a sensitivity of 45% (CI 36–54%) and a NPV of 93% (CI 91–95%). These results suggested that a negative ADXBLADDER result during surveillance was significantly associated with the absence of recurrence, conversely a positive result was not diagnostic.

### Oncuria

Furuya et al. identified 10 protein biomarkers (APOE, ANG, A1AT, CA9, IL8, MMP9, MMP10, PAI1, SDC1 VEGA) establishing a diagnostic signature (Oncuria™) for BC, which in a study including 44 subjects showed a sensitivity of 85% and specificity of 81% [68]. This test is currently under evaluation in several multicenter prospective study (NCT03193541, NCT03193528 and NCT03193515) and needs further extensive validation.

## 9. Non-Coding RNA

Non-coding RNA have been recognized as driver of BC carcinogenesis and progression [95,96,97,98,99], suggesting their potential uses as biomarkers for diagnosis and prognosis of bladder tumors [69,100,101]. Several mi-RNAs were reported as deregulated in BC and most of the studies investigated tissue samples [98,101]. However, Parizi et al. in a study including 157 subjects showed that miRNAs expression profile was different among urine samples from BC and healthy patients [70].

Hanke et al. for the first time reported the presence of miRNAs in urine in 2010 [71]. Since then, a lot of studies focused on the role miRNAs as urinary biomarkers, showing a potential prognostic ability for miR-200, miR-145, and miR-214 [70]. However, further studies on large population are needed to validate these findings.

LncRNAs affects cellular biology and are often deregulated in BC, suggesting their potential use as biomarkers in liquid biopsy samples for early diagnosis as well as for risk stratification [72]. As lncRNAs can be detected in urine and can resist to degradation by ribonuclease, several authors investigated their ability to detect or provide prognostic information in BC patients [73,74].

UCA1 is the most studied among lncRNAs as urinary biomarkers. A meta-analysis including seven studies showed that UCA1 has a good sensitivity (0.84) and specificity (0.87) for the detection of BC [75]. The combination of UCA1 with miR-210 and miR-96, and the mRNA, *HYAL1* achieved the ideal sensitivity of 100% with a specificity of 89.5 for BC detection [102]. Yazarlou et al. [69] measured the expression levels of four lncRNAs in urinary exosomes: UCA1-203, UCA1-201, MALAT1 and LINC00355 and the combined model showed a 92% sensitivity and a 91.7% specificity in a study population including 108 subjects. Based on their high sensitivity and specificity, lncRNAs seems to be extremely promising as urinary biomarkers. Further studies are encouraged to validate their clinical use and implement these assays into routine practice.

## 10. mRNA Signatures

### 10.1. Cxbladder Detect and Monitor

Cxbladder^®^ is a reverse transcription quantitative PCR assay designed to detect and quantify urinary mRNA levels of CDK1, MDK, HOXA13, IGFBP5 and CXCR2 [103]. Two versions of the assay are available: Cxbladder^®^ Detect and Cxbladder^®^ Monitor. The first showed high sensitivity and specificity (82% and 85%, respectively) in the detection of BC in subjects with hematuria [76]. Cxbladder^®^ Monitor showed high sensitivity and NPV to select patients at low risk of recurrence who would not benefit from repeated cystoscopy [77,78].

Koya et al. demonstrated that Cxbladder^®^ Monitor addition in clinical guidelines allowed to safely select patients who can be monitored by a cystoscopy every 2 years [79]. Lowering the number of cystoscopies, this test potentially showed the ability of decrease the economic burden of BC patient’s surveillance and increase patient compliance.

### 10.2. Xpert Bladder Cancer Detect and Monitor

The Xpert^®^ Bladder Cancer is a urinary test, which combines the values of five mRNA (*ABL1, ANXA10, CRH, IGF2 and UPK1B*) that may be overexpressed in BC by RT-PCR [80]. The test is performed using the Gene Xpert System (Cepheid) with an easy and fast assay, which automates nucleic acid amplification and the detection of mRNAs targets [80]. Two kind of assays have been developed: the first, detection, for first diagnosis, the second, monitor, for surveillance [81]. Xpert Detection showed a NPV of 99% (CI 98–100%) for high-grade cancers [81]. Several authors showed that active surveillance is a safe and cost-effective alternative therapeutic strategy in patients with recurrent NMIBC [82,83]. D’Elia et al. in a prospective study including 230 patients and 52 recurrences demonstrated that Xpert BC Monitor has a sensitivity of 85.7% for high-grade tumors [84]. Pichler et al. reported a high sensitivity (77%) also in low-grade disease [85]. In a recently published study, Xpert Bladder Monitor had an overall sensitivity of 74% with a specificity of 80%, and a sensitivity of 83% for high grade and 63% for low-grade cancers [86]. Interestingly, Hurle et al. reported the ability of Xpert Bladder Monitor to avoid unnecessary cystoscopies without missing high-grade cancers in a set of 106 patients underwent active surveillance when a cutoff value <0.4 was chosen [87].

## 11. Predict Targeted Therapy Efficacy

Urine can be collected after treatment as a source of biomarkers useful to assess residual disease or recurrence of BC. The staging of residual disease after neoadjuvant chemotherapy (NAC) might be ameliorated by sensitive detection of cancer genetic derivative. The staging allows us to select patients who achieve ypT0 for radical cystectomy avoidance. Unfortunately, no urine biomarkers are currently available to address this issue and the assessment of the minimal residual disease after NAC is still challenging.

The clinical scenario of BC is characterized by a high degree of heterogeneity high rate of recurrence and the limited treatment options [104], thus liquid biopsy in urine might represent a valuable source of predictive biomarkers for personalized medicine [105].

A panel of nine cytokines (tumor necrosis factor-a, tumor necrosis factor-related apoptosis-inducing ligand, IL-18, IL-12[p70], IL-12[p40], IL-8, IL-6, IL-2, IL-1ra) measured in urine has been used to build a nomogram (CyPRIT) able to predict recurrence after intravesical BCG with 85.5% accuracy [106]. More recently, Salmasi et al. demonstrated that SHGB, IP10 and resistin urinary levels were significantly associated with time-to-treatment failure in a prospective study including 50 patients receiving BCG (15 developed recurrence and 4 experienced disease progression) [107].

The test Uroseek [50] detect mutations in 11 genes, including PIK3CA, FGFR3 and ERBB2, which are target of drugs. Thus, this urinary test has the potential to guide treatment decision. FGFR3 mutations are preponderant in BC [91], thus FGFR3 inhibitors could be a desirable treatment option in patients who could be selected by urine biopsy.

Similarly, to what has been described in lung cancer [108], patients may be selected for kinase inhibitors treatment based on urine biopsy. Choudhury et al. demonstrated the efficacy of Afatinib in metastatic patients with cancers platinum-refractory presenting ERBB2 and ERBB3 mutations [109]. This drug could be given only in selected patients, potentially improving treatment outcome.

## 12. Conclusions and Future Perspective

Early identification of BC is a relevant goal. The five-year survival rate is about 94% when BC was early detected, but dramatically drops at 50% when the disease is muscle-invasive and at 20% in metastatic patients [110,111].

The currently used procedure for diagnosis and follow-up of BC relies on cystoscopy and urine cytology with substantial discomfort for the patient and high economic burden. Unfortunately, cytology has high specificity, but low sensitivity, so it is far from ideal for cystoscopy replacement. In this clinical scenario, a non-invasive and inexpensive biomarker could be useful. A biomarker with good sensitivity could replace cytology for the diagnosis, as well as a biomarker with high NPV could reduce repeated invasive cystoscopic procedures during the follow-up. In addition, a desirable outcome might be the availability of specific biomarkers to predict the response to targeted therapy.

For diagnostic purpose, few biomarkers are currently available with higher sensitivity, but lower specificity compared to cytology. In addition, some of them are not easy-to perform, making more complex to implement them in clinical practice [112]. The first biomarkers proposed were NMP22 [113] and BTA [114]. Unfortunately, since bladder tumors harbor different molecular changes, single biomarkers are far from ideal for BC detection. High-throughput technologies prompted new biomarkers discovery and panels building and several groups investigated the clinical benefit of multiplex assays (i.e., methylation of several genes and RNA signatures) [52,71,115,116,117,118].

For the follow-up, a restricted number of biomarkers are at present used in clinic. Only NMP22 and UroVysion are cited in EAU and AUA guidelines, but not widespread used in clinical settings.

To date, no biomarker is available to predict therapy response. However, considering that immunotherapeutic agents against programmed cell death protein 1 (PD-1) and programmed cell death ligand-1 (PD-L1) are emerging therapeutic options, predictive biomarkers could be particularly helpful.

The bladder is naturally pointed towards urine, which provides a source for liquid biopsy. Liquid biopsy has the potential to provide biomarkers for detection, prognosis, surveillance, monitoring the clinical outcome after treatment. Growing evidence suggest the need of clinical validation for the new discovered urinary biomarkers, potentially filling a relevant request for patient evaluation. Several challenges need to be addressed, such as low mutant allele fraction, tumor DNA stability in urine, the need of new technologies and correct storage and the transport of samples. Moreover, sequencing and detection of tumor DNA is still an expensive procedure and it could be cost-effective only if it can allow a change in therapy or the identification of druggable targets.

NMIBC shows high recurrence rate and cystoscopy currently is still the gold-standard diagnostic procedure, so clinical management of BC is one of the most expensive [119]. In the last ten years, assays based on panel of biomarkers were developed most of all for the follow-up. Several test, such as Urovysion, BTA and NMP22 are FDA-approved, but not widely used in clinical practice because of low NPV [55]. Xpert Bladder Monitor, Bladder EpiCheck and CxBladder Monitor showed more promising results with NPV higher than 98% [32,77,86].

Bladder tumors show cellular and molecular inter- and intra-tumoral heterogeneity [120,121]. Urine corresponds to a framework of the tumor heterogeneity, so mutations not detected by a biopsy procedure may be captured in urine. Thus, it is critical to develop urinary test able to address specific clinical need. The clinical validation has to focus only on test able to impact on clinical decision making process both in terms of improvement of patient survival and quality of life. Urine biopsy may represent a non-invasive test with the potential of significantly improve the whole diagnostic and therapeutic pathway of BC patient. Ideally, a multiplex assay could help to select which patients need invasive, expensive and time-consuming procedure such as cystoscopy, should predict disease recurrence and might envision the best treatment option for each single patient.

## Figures and Tables

**Table 1:** Current and upcoming liquid biopsy urinary tests.

Test	Variables	Method	Use in Clinical Practice	Cost	Approval	Reference
Bladder EpiCheck	DNA methylation pattern (15 genomic biomarkers)	RT-PCR	Diagnosis	NA	Yes (FDA/CE)	[27,28,29,30,31,32]
Urodiag	FGFR3 mutations and epigenetic markers (hypermethylation of HS3ST2, SEPTIN9 and SLIT2)	PCR	Diagnosis	NA	Yes (CE)	[33,34,35]
AssureMDX	FGFR3, TERT, HRAS mutations, methylation of OTX1, ONECUT2, TWIST1	PCR	Diagnosis	NA	No	[36,37]
UroMuTERT	TERT promoter mutations (C228T and C250T)	PCR	Diagnosis	NA	No	[38,39,40,41,42]
Uromonitor-V2	FGFR3, TERT and KRAS hotspot mutations	qPCR	Detection of NMIBC recurrence	NA	Yes (FDA/CE)	[43,44]
Urovysion	3,7,17 chromosome aneuploidy and loss of 9p21 locus	FISH	Diagnosis	200$/test	Yes (FDA/CE)	[45,46,47,48,49]
Uroseek^®^	Mutations in 11 genes and aneuploidy	NGS/SANGER	Diagnosis	1000$/test	No	[50,51]
uCAPP-Seq	Urine ctDNA determination	NGS	Diagnosis	NA	No	[52]
NMP22	Nuclear matrix protein released by dead cells in urine	ELISA	Surveillance in combination with cystoscopy	25$/test	Yes (FDA/CE)	[53,54,55]
BTA	Basement membrane-derived antigen released by cancer cells	ELISA	Surveillance in combination with cystoscopy	40$/test	Yes (FDA/CE)	[56,57,58,59]
ADXBLADDER	MCM5 protein	ELISA	Surveillance; prognosis	52$/test	Yes (CE)	[60,61,62,63,64,65,66,67]
Oncuria	10 protein biomarkers (APOE, ANG, A1AT, CA9, IL8, MMP9, MMP10, PAI1, SDC1, VEGFA)	ELISA	Diagnosis	NA	No	[68]
miRNAs	miR-200, miR-145, and miR-214	RT-qPCR	Prognosis	NA	No	[69,70,71]
LncRNA	UCA-1, UCA1-203, UCA1-201, MALAT1 and LINC00355	RT-qPCR	Diagnosis	NA	No	[72,73,74,75]
Cxbladder detect and monitor	mRNA signature in urine (CDK1, MDK, HOXA13, IGFBP5 and CXCR2)	RT-qPCR	Diagnosis, surveillance	NA	Yes (CE)	[76,77,78,79]
Xpert Bladder cancer detect and monitor	mRNA signature in urine (ABL1, ANXA10, CRH, IGF2 and UPK1B)	RT-qPCR	Diagnosis, surveillance	165$/test	Yes (CE)	[80,81,82,83,84,85,86,87]

Abbreviations: FDA: Food and Drug Administration; CE: Conformitè Europëenne; FGFR3: fibroblast growth factor receptor 3; HS3ST2: heparan sulfate-glucosamine 3-sulfotransferase 2; SLIT2: slit guidance ligand 2; TERT: telomerase reverse transcriptase; KRAS: Kirsten RAt sarcoma virus; NMIBC: non-muscle-invasive bladder cancer; ELISA: enzyme-linked immunosorbent assay; MCM5: minichromosome maintenance complex component 5; APOE: apolipoprotein E; ANG: angiogenin; A1AT: alpha-1 antitrypsin: CA9: carbonic anhydrase IX; IL8: interleukin-8, MMP9: metalloprotease-9, MMP10: metalloprotease-10, PAI1: plasminogen activator inhibitor 1, SDC1: Syndecan 1; VEGFA: vascular endothelial growth factor A; bps: base-pairs; RT-qPCR; real time quantitative polymerase chain reaction; NGS: next generation sequencing; CDK1: cyclin-dependent kinase 1, MDK: midkine, HOXA13: homeobox A13, IGFBP5: insulin like growth factor binding protein 5; CXCR2: CXC chemokine receptor 2; ABL1: tyrosine-protein kinase; ANXA10: annexin A10; CRH: corticotropin-releasing hormone, IGF2: insulin like growth factor 2; UPK1B: uroplakin 1B, NA: not available.

## Supplementary Materials

The following are available online at https://www.mdpi.com/2075-4426/11/3/237/s1, Figure S1: Study selection flow chart.

## Abbreviations

FDA: food and drugs administration; CE: Conformitè Europëenne; FGFR3: Fibroblast Growth Factor Receptor 3; HS3ST2: Heparan Sulfate-Glucosamine 3-Sulfotransferase 2; SLIT2: Slit Guidance Ligand 2; TERT: Telomerase reverse transcriptase; KRAS: Kirsten RAt Sarcoma virus; NMIBC: nonmuscle-invasive bladder cancer; ELISA: Enzyme-linked immunosorbent assay; MCM5: Minichromosome Maintenance Complex Component 5; APOE: Apolipoprotein E; ANG: Angiogenin; A1AT: Alpha-1 antitrypsin: CA9: Carbonic anhydrase IX; IL8: Interleukin-8, MMP9: metalloprotease-9, MMP10: metalloprotease-10, PAI1: plasminogen activator inhibitor 1, SDC1: Syndecan 1; VEGFA: Vascular endothelial growth factor A; bps: base-pairs; RT-qPCR; real time quantitative polymerase chain reaction; NGS: next generation sequencing; CDK1: Cyclin-dependent kinase 1, MDK: Midkine, HOXA13: Homeobox A13, IGFBP5: Insulin Like Growth Factor Binding Protein 5; CXCR2: CXC chemokine receptor 2; ABL1: Tyrosine-protein kinase; ANXA10: Annexin A10; CRH: Corticotropin-releasing hormone, IGF2: Insulin Like Growth Factor 2; UPK1B:Uroplakin 1B, NA: not available. BC: bladder cancer; NMIBC, non-muscle invasive bladder cancer; MIBC, muscle-invasive bladder cancer; RT-PCR, real-time polymerase chain reaction; miRNA, micro-RNA; LncRNA, long-non coding-RNA.
