# Supplementary figures and images for: Liquid Biopsy Biomarkers in Urine: A Route towards Molecular Diagnosis and Personalized Medicine of Bladder Cancer
Source: J Pers Med. 2021 Mar 23;11(3):237. doi: 10.3390/jpm11030237 (PMC8004687; doi:10.3390/jpm11030237)

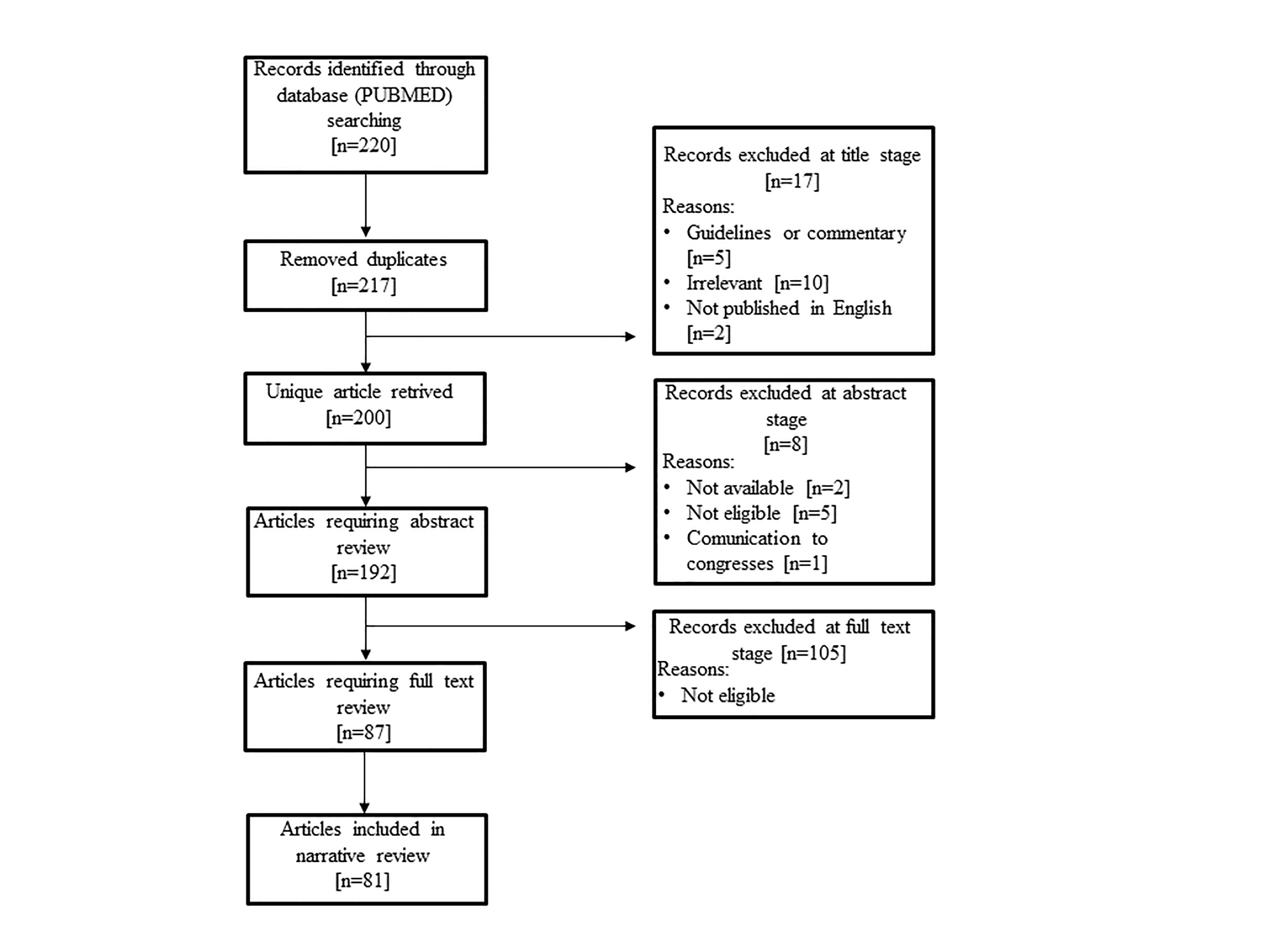

Supplement: Supplementary file 1 [file jpm-11-00237-s001.zip › jpm-1109133-supplementary.tif]
